# Lysine acetylome profiling reveals a dual regulatory role in carbon flux redirection and RNA degradation inhibition in *Mycobacterium smegmatis*

**DOI:** 10.3389/fmicb.2026.1823773

**Published:** 2026-05-29

**Authors:** Manluan Sun, Qiyu Zhao, Caiquan Zhou, Chenghao Liu, Bingyu Yang, Jia Bu, Ruilan Li, Jiang Bian, Xiaojie Niu, Jinbo Hu, Binyu Liu, Sai Ge

**Affiliations:** 1 School of Medicine, Shanxi Datong University, Datong, China; 2 Institute of Medical Microecology and Drug Discovery and Development, Shanxi Datong University, Datong, China; 3 Department of Plant Biology, Swedish University of Agricultural Sciences, Uppsala, Sweden; 4 Center of Academic Journal, Shanxi Datong University, Datong, China

**Keywords:** 7H9 medium, lysine acetylation, *Mycobacterium smegmatis*, post-translational modification, Sauton medium

## Abstract

**Introduction:**

*Mycobacterium tuberculosis* in natural environments and host organisms must adapt to constantly changing growth conditions, and its adaptive mechanism for nutrient metabolism represents one response to complex environments. Reversible post-translational protein modifications regulate central metabolic enzymes in *M. tuberculosis*, thereby governing its adaptation to varying environmental nutrient availability.

**Methods:**

In this study, we cultured *Mycobacterium smegmatis* MC^2^155 using Sauton or Middlebrook 7H9 media and applied liquid chromatography–tandem mass spectrometry (LC–MS/MS) to analyze differences in acetylation-modified proteins. We also performed bioinformatic analysis of the acetylated proteins expressed in the different media.

**Results:**

LC–MS/MS revealed 182 acetylated proteins and 398 sites exclusively in Sauton-medium-cultured strains; whereas 57 acetylated proteins and 141 sites were identified exclusively in nutrient-rich 7H9-medium-cultured strains. Additionally, 302 proteins and 462 sites were differentially acetylated between the Sauton- and 7H9-medium-cultured samples. Our bioinformatics analysis identified differences in whole-protein acetylation modifications in *M. smegmatis* MC^2^155 under these two culture conditions, primarily reflected in metabolic pathways, including the citrate cycle (TCA cycle), 2-oxocarboxylic acid metabolism, carbon metabolism, RNA degradation, and tryptophan metabolism.

**Discussion:**

Under the nutrient-limited conditions of Sauton medium culture, multiple sites within isocitrate dehydrogenase exhibited acetylation, leading to reduced enzyme activity. This effect may redirect a greater proportion of carbon flux towards the glyoxylate pathway. Conversely, in 7H9 medium, acetylation at residues K189 and K331 of isocitrate lyase may diminish enzyme activity, thereby channeling increased carbon flux towards the TCA cycle. Acetylation at 3-hydroxyacyl-CoA dehydrogenase (K370) and tryptophan-tRNA synthetase (K200) may reduce fatty acid and protein synthesis, thereby preventing excessive energy expenditure; acetylation at Oligoribonuclease K153 likely diminishes enzyme activity, thereby allowing *M. smegmatis* to adapt to nutrient limitation by accumulating more c-di-AMP.

## 1 Introduction

Infectious disease tuberculosis (TB), caused by *M. tuberculosis*, remains one of the most serious public health threats worldwide (Lukas et al., 2025). The World Health Organization (WHO) estimates that 10.7 million people contracted TB in 2024 (World Health Organization, 2025). The metabolic plasticity of *M. tuberculosis* is considered a key factor in human infection, prolonged latency within the host, evasion of immune clearance, and ultimate progression to TB (Lee et al., 2017; Borah et al., 2021; Bei et al., 2024; de Carvalho et al., 2010; Liu Y. et al., 2024). In humans, *M. tuberculosis* primarily resides within the nutritionally restricted environment of macrophage phagocytic lysosomes; however, it is also occasionally found in nutritionally rich environments, such as necrotic tissue, lymph, and blood (de Carvalho et al., 2010). This formidable adaptability stems from *M. tuberculosis*’s capacity to efficiently reprogram its metabolic pathways under diverse environmental conditions, thereby accommodating varying nutritional states (de Carvalho et al., 2010; Grigorov et al., 2025; Farnia et al., 2026).

Lysine acetylation (Kac) is one of the most prevalent post-translational protein modifications in both eukaryotes and prokaryotes, occurring on the *ε*-amino group of lysine side chains (Huang Y. et al., 2023; Xia et al., 2020). Lysine acetylation is widespread across many cellular processes, including transcription, phase separation, autophagy, mitosis, differentiation, and neural function (Shvedunova and Akhtar, 2022). Extensive recent studies in acetylated proteomics have demonstrated the prevalence and significance of acetylation modifications in prokaryotes. Fang et al. identified 1,039 acetylated proteins in wild-type *E. coli*, involving 2,971 lysine acetylation sites, and found that acetylation modifications could influence bacterial antibiotic resistance (Fang et al., 2022). Lozano-Terol et al. (2024) compared acetylation patterns in *E. coli* proteins across different carbon sources, media types, and growth stages. They observed distinct acetylation levels in GapA, Mdh, and AceA, which regulate central metabolic carbon flux, under contrasting culture conditions (restricted versus nutrient-rich media).

Post-translational protein modifications also contribute to *M. tuberculosis*’s metabolic plasticity (Huang Y. et al., 2023). Reversible post-translational modifications regulate key *M. tuberculosis* proteins, enabling rapid, flexible cellular responses to environmental stresses, and global modulation of the direction and rate of intracellular metabolism across varying cultural conditions. Lysine acetylation, which is reversible, plays a critical role in *M. tuberculosis*’s adaptation to environmental shifts (Huang Y. et al., 2023), virulence (Di et al., 2023), drug resistance (Sun et al., 2023), and nutritional metabolism (Farnia et al., 2026; Kan et al., 2024). For example, acetylation of the virulence factor EsxA enhances its binding to host cell membranes, facilitating mycobacterial cytoplasmic translocation and increased virulence (Aguilera et al., 2020). Moreover, acetylation of pknH attenuates *M. tuberculosis* resistance to ethambutol (Sun et al., 2023). Regarding adaptation to environmental challenges, studies have indicated that *M. tuberculosis* reduces CRP K193 acetylation to enhance CRP DNA binding and transcriptional activity in response to microenvironmental signals, such as cyclic adenosine monophosphate (cAMP), low pH, elevated temperature, and oxidative stress. This effect amplifies CRP’s role as a global regulator, facilitating stress adaptation (Di et al., 2023). The acetyltransferase Eis and NAD+ -dependent deacetylase Rv1151c regulate the acetylation status of the histone-like NAP HupB in response to stress (Green et al., 2018). Meanwhile, acetylation modifications of ICL and ICDH modulate carbon flux towards the TCA cycle and glyoxylate pathway (Lee et al., 2017; Farnia et al., 2026; Huang E. Y. et al., 2023).

Despite extensive research on *M. tuberculosis*, many regulatory mechanisms under varying nutritional conditions remain unresolved. For instance, the mechanisms governing carbon flux regulation under different nutritional conditions and how transcriptional plasticity enables adaptation to environmental stressors under such conditions remain poorly known (Borah et al., 2021; Bei et al., 2024). In the present study, we investigate the acetylation regulatory mechanisms of mycobacteria under varying nutritional conditions to characterize their metabolic plasticity. *M. smegmatis* is often used as an ideal model system for *M. tuberculosis* because of its non-pathogenicity, fast growth, and similarity to *M. tuberculosis* in most cellular profiles. It is necessary to conduct systematic research on this model mycobacteria to further our understanding of TB. Thus, we performed comparative proteomics in *M. smegmatis* cultured under different nutritional conditions to better understand the differences in protein abundance and the biological significance of lysine acetylation (Kac) in this strain. *M. smegmatis* strains were cultured in either Middlebrook 7H9 or Sauton liquid media. 7H9 medium, which is relatively nutrient-rich and contains Tween 80, allows *M. smegmatis* to grow in a dispersed state with ample oxygen, simulating a nutrient-rich environment. In contrast, Sauton medium relies solely on asparagine as its primary nitrogen source and glycerol as its main carbon source, presenting a nutritionally deficient environment. The absence of surfactants in Sauton medium promotes the aggregated growth of *M. smegmatis* under microaerophilic conditions, simulating nutrient-limited conditions (Zhang L. Y. et al., 2022). The lysine acetylome was determined using label-free liquid chromatography–tandem mass spectrometry (LC–MS/MS); 603 acetylated sites on 359 proteins were identified in *M. smegmatis* cultured in Middlebrook 7H9 medium, and 860 acetylated sites on 484 proteins in *M. smegmatis* cultured in Sauton liquid medium. Among these proteins, 302 showed differential site or abundance between the cells grown in different media.

## 2 Materials and methods

### 2.1 *Mycobacterium smegmatis* strains and culture conditions

Take the activated *M. smegmatis* MC2155 strain and culture it in 7H9 liquid medium until the mid-log phase. A single subculture was used to obtain a stable experimental strain. Subsequently, the resultant strain is inoculated at a dilution ratio of 1:100 into two distinct culture media: Middlebrook 7H9 broth with 10% OADC medium enrichment and 0.05% Tween 80 and Sauton liquid media (BD, Maryland, USA) (Venkataswamy et al., 2012). For the preparation of 1 L Sauton liquid medium, 4.0 g/L L-asparagine monohydrate, 2.8 g/L trisodium citrate dihydrate, 0.5 g/L dipotassium hydrogen phosphate, 0.5 g/L magnesium sulfate heptahydrate, 0.05 g/L ferric ammonium citrate, and 6% (v/v) glycerol were used (Yamaguchi et al., 2025). The strains.were incubated at 37 °C to the mid-log phase for subsequent experimental use.

### 2.2 Protein extraction and sample preparation

The bacterial cells cultured in two different media were harvested by centrifugation at 8,000 × *g* for 10 min at 4 °C, respectively. The cells were then washed twice with chilled phosphate-buffered saline (PBS) and centrifuged at 12,000 × *g* for 5 min. The pellets were resuspended in 50 mL of ice-cold lysis buffer [50 mM Tris–HCl [pH 7.5], 100 mM NaCl, 5 mM dithiothreitol (DTT), and 50 mM nicotinamide (Liu et al., 2014)], and sonicated. Cell debris was removed by centrifugation at 12,000 × *g* for 30 min at 4 °C. Protein concentration was determined by using the Bradford Protein Assay Kit (Bio-Rad, Hercules, USA).

### 2.3 Protease digestion in solution

DTT (25 mM) was added to 30 mg of bacterial total protein. Disulfide bonds were then reduced by incubation at 37 °C for 45 min iodoacetamide (50 mM) was added at 25 °C and the alkylation reaction was conducted for 20 min in the dark. Excess iodoacetamide was quenched by adding DTT before trypsin digestion. Subsequently, trypsin was added to the protein sample at a 1:100 (w/w) ratio, and the sample was incubated at 37 °C for 4 h, followed by a 20 h incubation at 37 °C in trypsin solution at the same ratio. Trypsin digestion was quenched with 0.1% trifluoroacetic acid (TFA), and peptides were desalted using a C18 column. Finally, the sample was eluted with 75% acetonitrile (0.1% TFA) and dried in a SpeedVac.

### 2.4 Enrichment of lysine acetylated peptides

Lysine-acetylated peptides were enriched using the PTMScan® Acetyl-Lysine Motif [Ac-K] Kit (Cell Signaling Technology, CST, Cat. No. 13416) following the manufacturer’s instructions. Briefly, dried tryptic peptides were dissolved in 1 × IAP buffer, cleared by centrifugation at 10,000 × g for 5 min at 4°C, and incubated with pre-washed acetyl-lysine motif antibody-conjugated beads for 2 h at 4 °C with rotation. The beads were washed twice with 1 × IAP buffer and three times with chilled HPLC-grade water. Acetylated peptides were eluted twice with 0.15% TFA, combined, desalted using C18 tips or StageTips, and subjected to LC–MS/MS analysis.

### 2.5 LC–MS/MS analysis

Enriched acetylated peptides were analyzed using a Thermo EASY-nLC system coupled online to a Q Exactive Orbitrap mass spectrometer. Peptides were loaded onto the column with a loading volume of 15 μL at a flow rate of 4.0 μL/min and separated on a C18 reverse-phase analytical column at a constant flow rate of 300 nL/min. Mobile phase A consisted of 0.1% formic acid in water, and mobile phase B consisted of 0.1% formic acid in acetonitrile. The LC gradient was set as follows: 0–2 min, 0–6% B; 2–105 min, 6–23% B; 105–130 min, 23–29% B; 130–147 min, 29–38% B; 147–148 min, 38–48% B; 148–149 min, 48–100% B; 149–155 min, 100% B; 155–156 min, 100–0% B; and 156–160 min, 0% B for column equilibration. The Q Exactive Orbitrap mass spectrometer was operated in positive ion mode using a data-dependent acquisition method. Full MS scans were acquired in the Orbitrap over an m/z range of 350–1300 at a resolution of 70,000, with an AGC target of 3 × 10⁶ and a maximum injection time of 20 ms. The top 20 most intense precursor ions were selected for higher-energy collisional dissociation fragmentation. MS/MS spectra were acquired at a resolution of 17,500, with an AGC target of 1 × 10⁵, a maximum injection time of 80 ms, an isolation window of 2.0 m/z, and a normalized collision energy of 30. The MS/MS scan range was m/z 200–2000, with a fixed first mass of m/z 100. Singly charged, unassigned, and highly charged precursor ions were excluded from fragmentation. Dynamic exclusion was enabled with an exclusion duration of 30 s. Raw data were processed as described below.

### 2.6 Data processing

Raw MS/MS data were processed using MaxQuant version 2.0.1.0. Spectra were searched against the NCBI RefSeq protein database of *Mycobacterium smegmatis* str. MC2 155, containing 6,717 protein sequences. A reverse decoy database was generated automatically by MaxQuant for false discovery rate estimation. Trypsin was selected as the digestion enzyme, and up to two missed cleavages were allowed. Carbamidomethylation of cysteine residues was set as a fixed modification, while methionine oxidation and lysine acetylation were set as variable modifications. The precursor ion mass tolerance was set to 4.5 ppm, and the fragment ion mass tolerance was set to 20 ppm. Peptides shorter than six amino acids were excluded from the analysis. The false discovery rate was controlled at 1% for peptide and protein identification.

### 2.7 Bioinformatics analysis

Using the MEME suite in MoMo software with the motif-x algorithm, we selected the most significant position-residue pairs to identify motifs. The 15 amino acid residues upstream and downstream of each Kac site were analyzed (Cheng et al., 2019).

We performed functional annotation and enrichment analyses of acetylated proteins differentially expressed or absent in *M. smegmatis* MC^2^155 cultured in Sauton or 7H9 medium. Gene Ontology (GO) analysis of the identified acetylated proteins was conducted using Blast2GO, covering the ontology categories cellular component (CC), molecular function (MF), and biological process (BP). Pathway analysis of the identified acetylated proteins was performed using the Kyoto Encyclopedia of Genes and Genomes (KEGG) metabolic pathway database.^1^ GO enrichment analysis was conducted using the Cytoscape plugin BiNGO (Maere et al., 2005), with the entire genome annotation of *M. smegmatis* as the background reference set.

The subcellular localization of acetylated proteins was determined using the PSORTb Subcellular Localization Prediction Tool, version 3.0.3^2^ (Yu et al., 2010). The settings used were as follows: Organism type = Bacteria, Gram stain = Positive, Output format = Short Format (tab-delimited), and Show result = Via the web. FASTA sequences of proteins retrieved from NCBI were input. Statistical analyses were conducted to determine the percentage of acetylated proteins localized to the cytoplasm, cell membrane, and cell wall.

Interactions among acetylated proteins were analyzed using the STRING database of functional protein–protein association networks (v12.0; https://cn.string-db.org/) and the Cytoscape 3.10.3 plugin MCODE (Shannon et al., 2003). In the STRING database list, we selected “Multiple proteins”, *M. smegmatis* as the organism, and subsequently entered the protein names of interest in the List Of Names field.

Subsequently, we used the Uniprot database to identify and analyze the functions of lysine acetylation sites in the identified acetylated proteins. Conservative analysis of these sites was performed using MEGA 12 software and ESPript 3.0 (Gouet et al., 2003). The raw data has been uploaded to iProX under login ID: IPX0016996000 (Ma et al., 2019; Chen et al., 2022).

## 3 Results

To compare the characteristics of bacterial protein acetylation under different nutritional conditions, we cultured *M. smegmatis* MC^2^155 in either Sauton or 7H9 medium. A quantitative analysis of acetylation sites in *M. smegmatis* MC^2^155 cultured these two media produced three datasets: (1) Sauton-specific acetylated proteins, which contain acetylation sites expressed exclusively after culturing in Sauton medium; (2) 7H9-specific acetylated proteins, which contain acetylation sites expressed exclusively after culturing in 7H9 medium; and (3) medium-differential acetylated proteins, which contain acetylation sites present after culturing in either Sauton or 7H9 media but exhibit differential acetylation signal intensity. Notably, these three categories of acetylated proteins overlap. For example, the acetylation levels of K120, K146, and K154 in transcription termination factor Rho differ between *M. smegmatis* MC^2^155 cultured in Sauton medium and that cultured in 7H9 media; K138 is acetylated only in *M. smegmatis* MC^2^155 cultured in Sauton medium; and K349 is acetylated only in *M. smegmatis* MC^2^155 cultured in 7H9 medium.

### 3.1 Identification of acetylation sites

**Figure 1:**
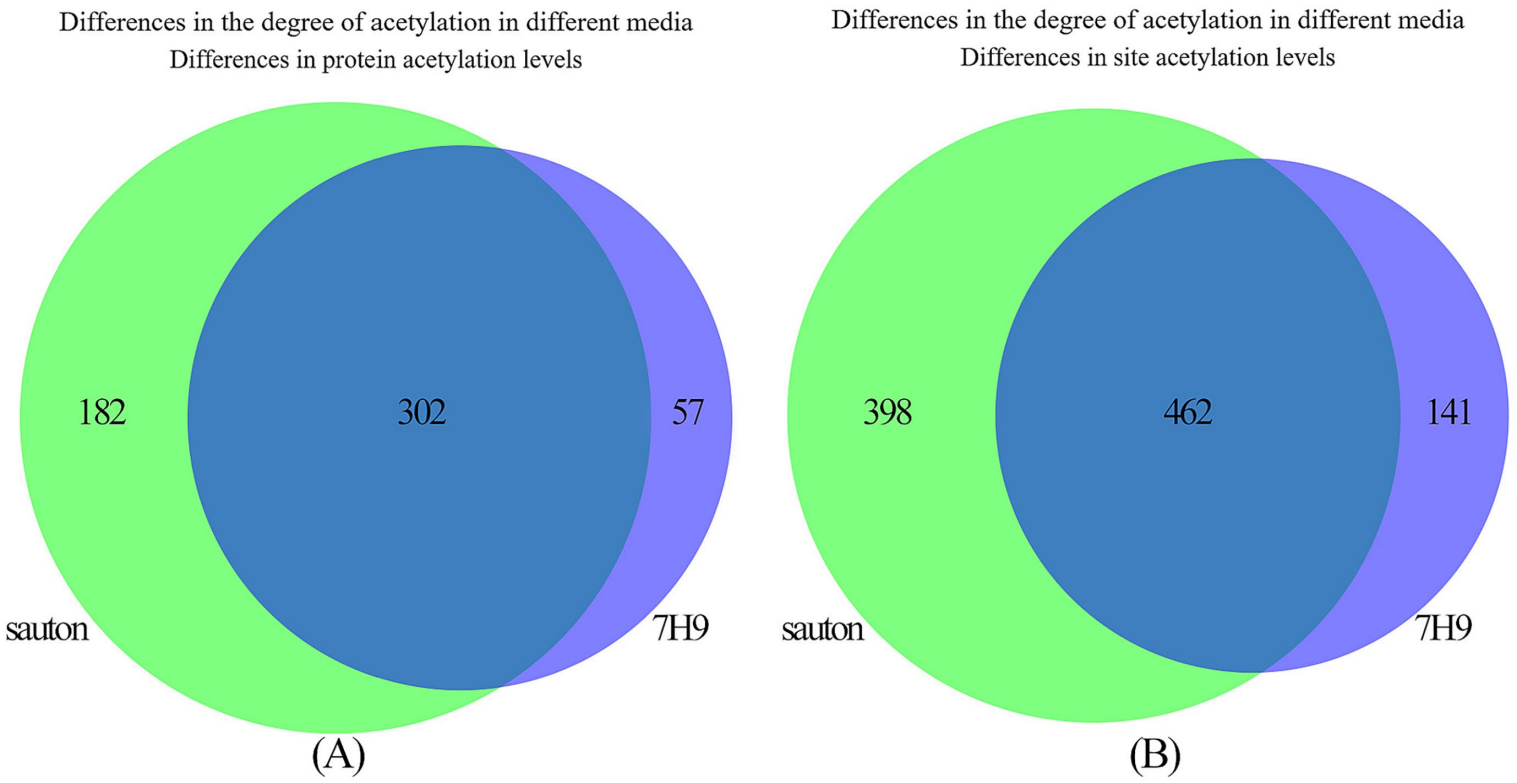
Expression differences of acetylated proteins and sites in different media. **(A)** Differential expression of acetylated proteins. **(B)** Differential expression of acetylation sites.

We identified 484 acetylated proteins and 860 acetylation sites in *M. smegmatis* MC^2^155 cultured in Sauton medium, and 359 acetylated proteins and 603 acetylation sites in *M. smegmatis* MC^2^155 cultured in 7H9 medium (Supplementary Table S1). The abundance of acetylated proteins and sites was significantly higher when *M. smegmatis* MC^2^155 was cultured in Sauton medium versus 7H9 medium (Figure 1). Furthermore, compared to 7H9-specific acetylated proteins, Sauton-specific acetylated proteins exhibited a higher abundance of acetylation sites. Among the Sauton-specific acetylated proteins, PspA/IM30 family protein, glucose-6-phosphate dehydrogenase (coenzyme-F420), DNA-directed RNA polymerase subunit beta’, and multifunctional oxoglutarate decarboxylase contained four acetylation sites, whereas chaperonin GroEL (TBH46394.1) contained five. In contrast, among the 7H9-specific acetylated proteins, only succinate-CoA ligase subunit alpha and 50S ribosomal protein L1 contained three acetylation sites, with the rest containing one or two.

The quantitative analysis of acetylation intensity revealed that, among the medium-differential acetylated proteins, 263 exhibited higher acetylation signal intensity at 416 sites in *M. smegmatis* MC^2^155 cultured in Sauton medium. Among these proteins, eight contained five acetylation sites. DNA-directed RNA polymerase subunit beta (TBH31865.1), elongation factor G, and sulfurtransferase (TBH48777.1) each contained six acetylation sites; type I DNA topoisomerase contained seven acetylation sites; and chaperonin GroEL (TBH46394.1) contained 11 acetylation sites.

### 3.2 Motif analysis

**Figure 2:**
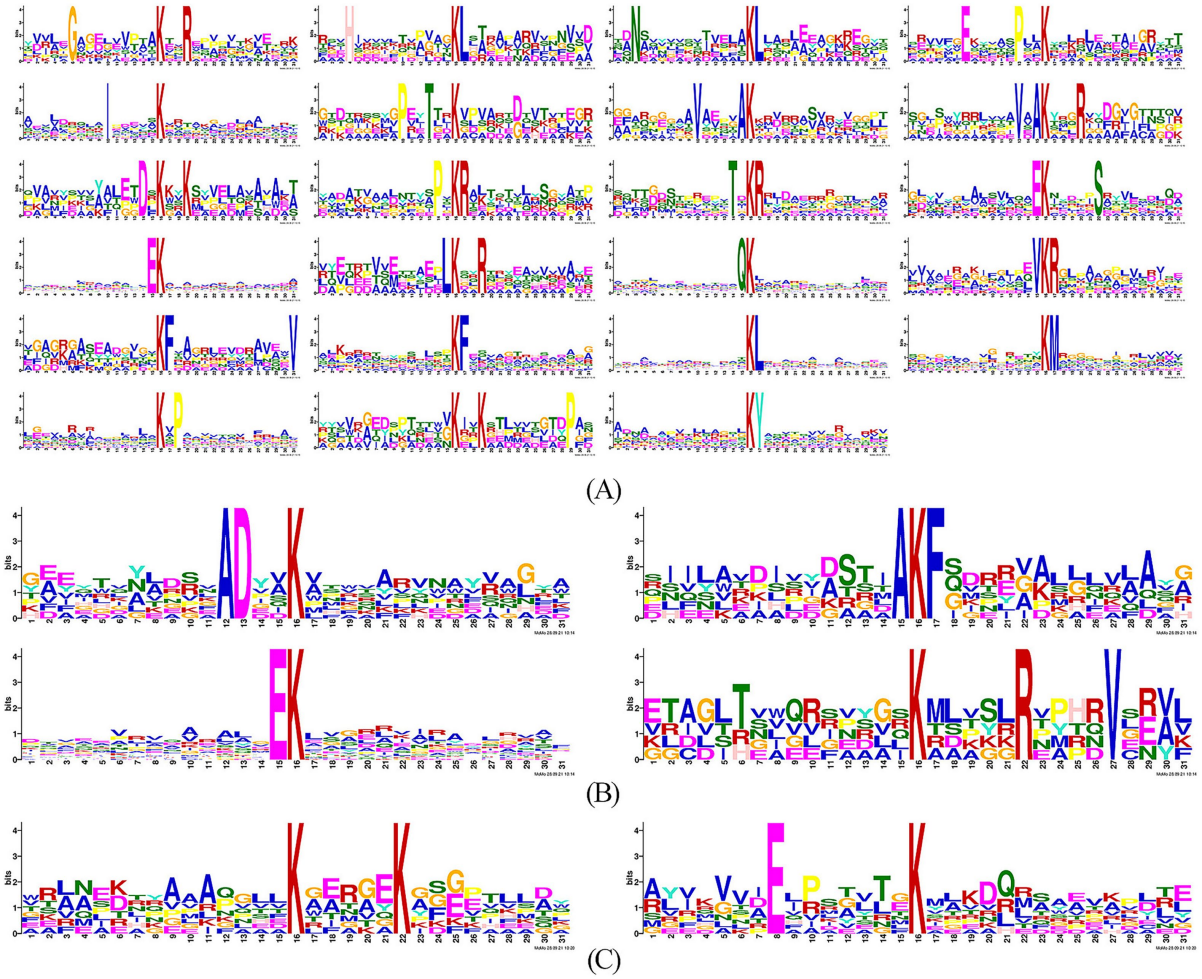
Motif analysis of acetylated proteins in *M. smegmatis* MC2 155. **(A)** Repeated motifs in Sauton-specific acetylated proteins. **(B)** Repeated motifs in 7H9-specific acetylated proteins. **(C)** Repeated motifs in medium-differential acetylated proteins.

We conducted motif analysis of the flanking amino acid sequences in MoMo to understand the distribution of amino acids surrounding lysine acetylation sites and reveal acetyltransferase preferences under different nutritional conditions (Supplementary Table S2). The motifs of Sauton-specific acetylated proteins exhibited greater diversity than those of 7H9-specific acetylated and medium-differential acetylated proteins. Twenty-three motifs were identified in Sauton-specific acetylated proteins: N*Kac L, H*Kac L, G*Kac*R, E*P*Kac, I*Kac, P*T*Kac, V*A Kac, V*A Kac*R, D*Kac*K, P*Kac R, T*Kac R, E Kac*S, E Kac, L Kac*R, Q Kac, V Kac R, Kac F*V, Kac F, Kac L, Kac M, Kac*P, Kac*K*P, and Kac Y. Four motifs were identified in 7H9-specific acetylated proteins: Kac*R*V, E Kac, A D*Kac, and A Kac F. Two motifs (E*Kac and Kac*K) were present in medium-differential acetylated proteins (see Figure 2).

Sauton-specific, 7H9-specific, and medium-differentiated acetylated proteins were found to feature acetylated lysine residues downstream of positively charged residues, such as lysine (K) and arginine (R), with negatively charged residues such as glutamic acid (E) and aspartic acid (D) found upstream. Xie et al. (2015) obtained similar results.

### 3.3 Functional classification and subcellular localization

Sauton medium is nutritionally restricted, with asparagine as the primary amino acid and glycerol as the principal carbon source, without surfactants, enabling *M. smegmatis* to aggregate and grow under microhypoxic conditions. 7H9 medium contains multiple carbon and nitrogen sources. To further investigate how environmental differences in protein acetylation affect *M. smegmatis* CC, MF, and BP categories, as well as metabolic pathways, we performed GO enrichment (Supplementary Table S3) and KEGG pathway analyses (Supplementary Table S4) on the identified proteins.

**Figure 3:**
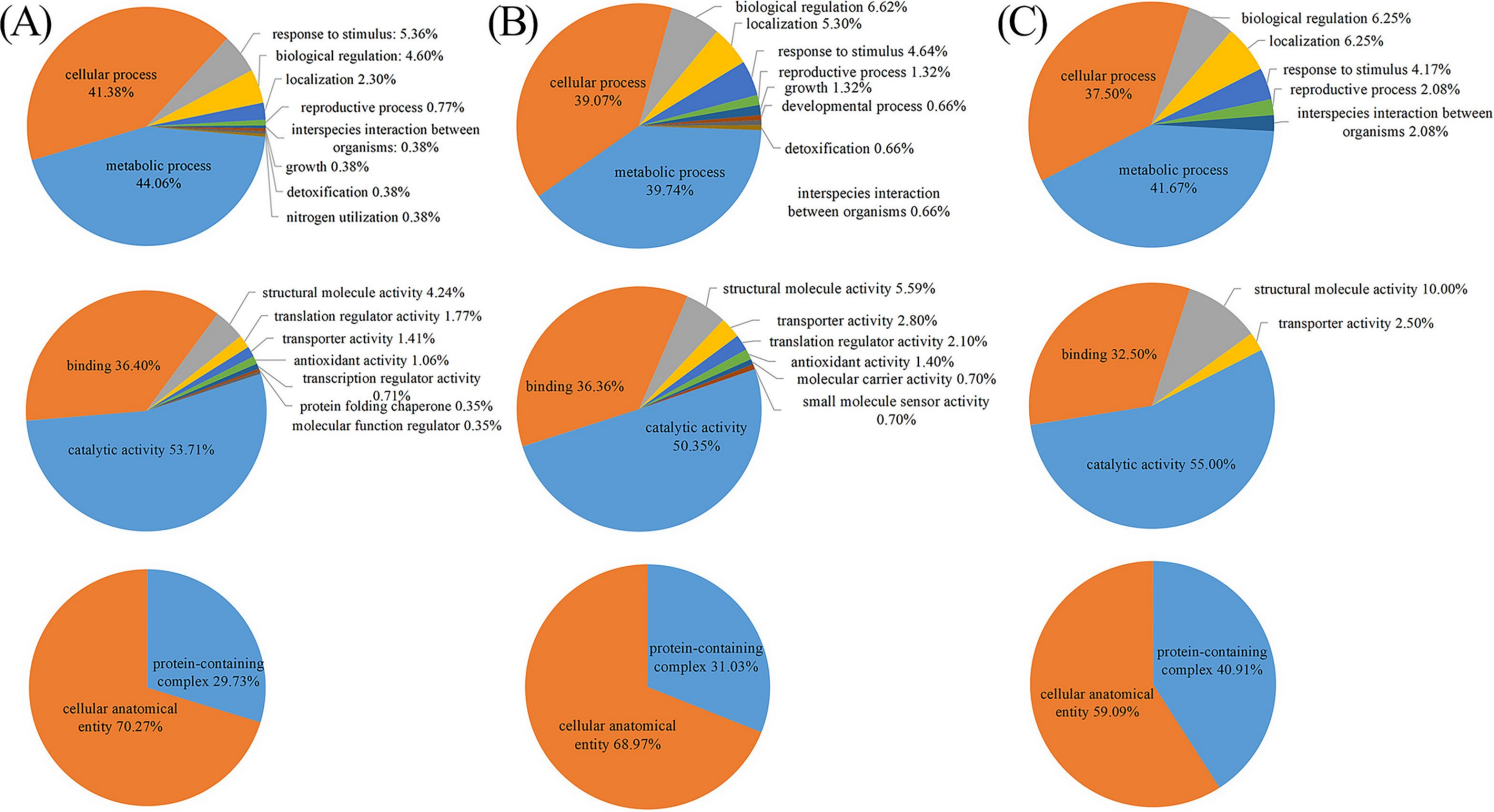
GO classification of acetylated proteins in *M. smegmatis* MC2 155. **(A)** Biological process, molecular function, cellular component of Sauton-specific acetylated proteins. **(B)** Biological process, molecular function, cellular component of 7H9-specific acetylated proteins. **(C)** Biological process, molecular function, cellular component of medium-differentially acetylated proteins.

GO enrichment analysis revealed differences in CC, MF, and BP categories between the Sauton-specific and 7H9-specific acetylated proteins (Table 1). However, their primary components were largely similar. For example, acetylated proteins involved in metabolic processes constituted approximately 44.06% of the BP categories among the Sauton-specific acetylated proteins (Figure 3A). Among the 7H9-specific and medium-differential acetylated proteins, this category constituted approximately 39.74% (Figure 3B) and 41.67% (Figure 3C), respectively.

**Table 1:** GO domains of various proteins.

GO domains	Similarities	Differences		
		Sauton-specific acetylated proteins	7H9-specific acetylated proteins	Medium-differential acetylated proteins
Biological process	Biological regulation Metabolic process Reproductive process Cellular process Interspecies interaction Localization response to stimulus	Growth Detoxification Developmental process	Growth Getoxification Nitrogen utilization	—^a^
Cellular component	Protein-containing complex Cellular anatomical entity	—^a^	—^a^	—^a^
Molecular function	Structural molecular activity Transporter activity binding Catalytic activity	Translation regulatory activity Antioxidant activity Molecular function regulatory Protein folding chaperone Transcription regulatory activity	Translation regulatory activity Antioxidant activity Molecular carrier activity Small molecule sensor activity	—^a^

a No differences in biological process, cellular component, or molecular function.

**Figure 4:**
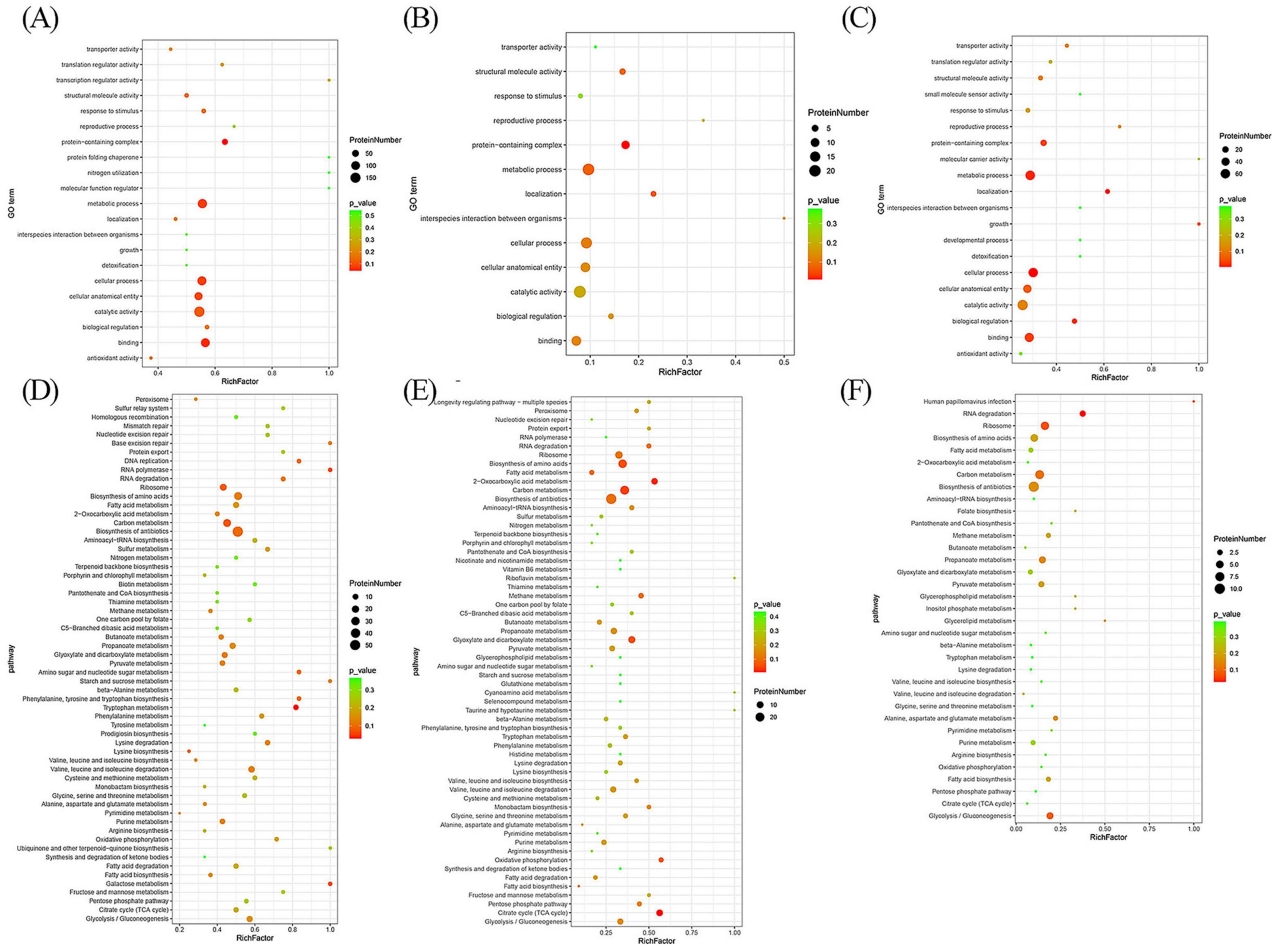
GO and KEGG analysis of acetylated proteins in *M. smegmatis* MC2 155. **(A)** GO analysis of Sauton-specific acetylated proteins. **(B)** GO analysis of medium-differential acetylated proteins. **(C)** GO analysis of 7H9-specific acetylated proteins. **(D)** KEGG analysis of Sauton-specific acetylated proteins. **(E)** KEGG analysis of 7H9-specific acetylated proteins. **(F)** KEGG analysis of medium-differential acetylated proteins.

Proteins constituting the protein-containing complex were significantly enriched among Sauton-specific (Figure 4A) and medium-differential (Figure 4B) acetylated proteins. Conversely, among 7H9-specific acetylated proteins, enrichment was observed in BPs such as cellular processes, localization, biological regulation, and metabolic processes, MFs such as binding, and CCs comprising protein-containing complexes (Figure 4C).

KEGG metabolic pathway analysis revealed that proteins associated with tryptophan metabolism were significantly enriched in Sauton-specific acetylated proteins (Figure 4D). In 7H9-specific acetylated proteins, those involved in metabolic pathways, including the citrate cycle (TCA cycle), 2-oxocarboxylic acid metabolism, and carbon metabolism, were significantly enriched (Figure 4E). Among medium-differential acetylated proteins, those involved in the RNA degradation pathway were significantly enriched (Figure 4F).

Furthermore, as subcellular localization of proteins correlates with amino acid sequences, their localization can be inferred from their sequences to reflect associated protein functions and metabolic pathways. Therefore, we employed the PSORTb Subcellular Localization Prediction Tool (version 3.0, https://psort.org/psortb/) to predict the subcellular localization of acetylated proteins. Among Sauton-specific acetylated proteins, 7H9-specific acetylated proteins, and medium-differential acetylated proteins, the proportions localizing to the cytoplasm, cell membrane, and cell wall were 75.50%/8.72%/1.01, 71.65%/11.02%/1.57, and 78.45%/6.71%/0.35% (Supplementary Table S5), respectively. The differences among the three groups were not significant.

### 3.4 Analysis of acetylated protein interactions

**Figure 5:**
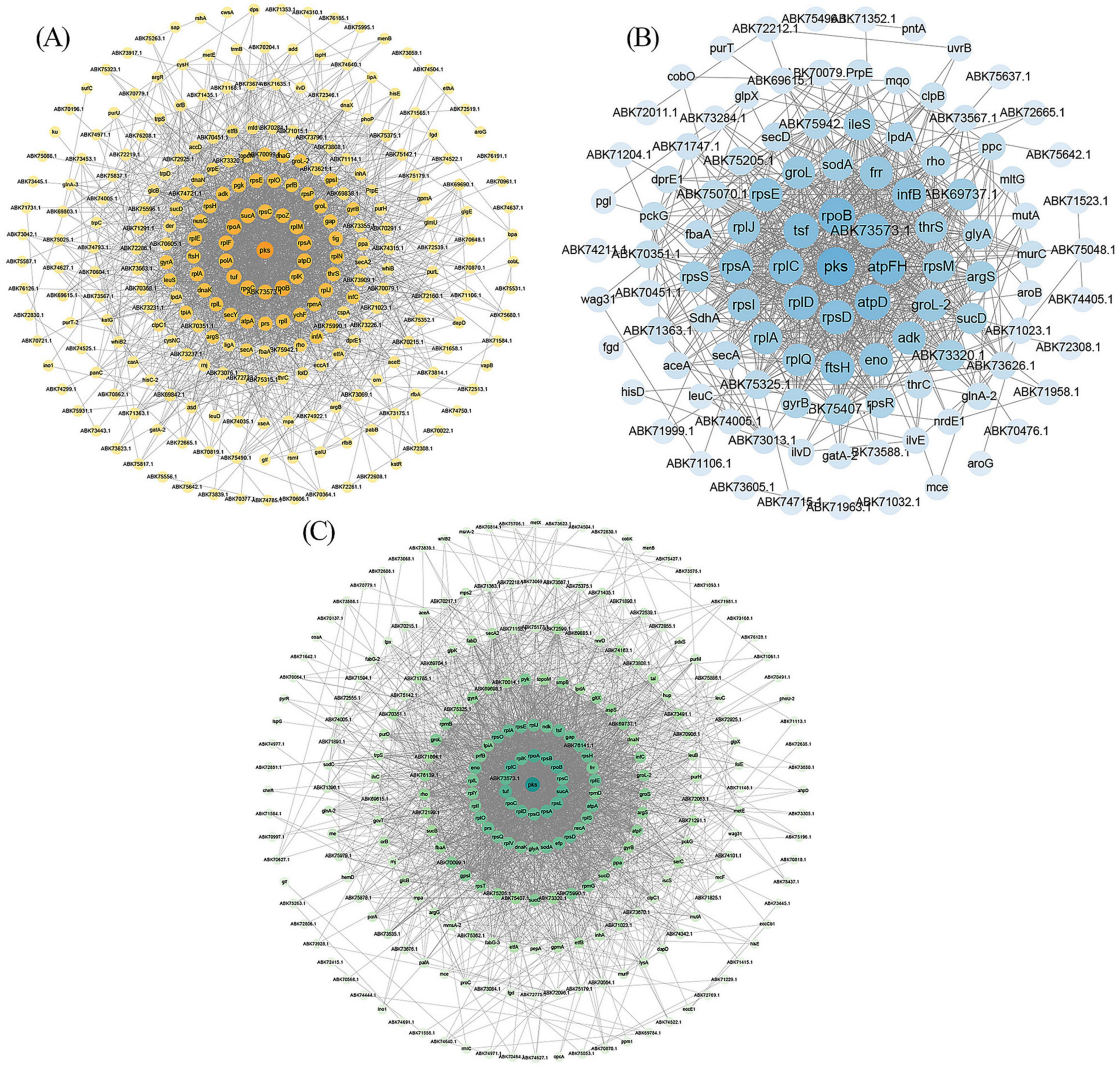
Protein interaction analysis of acetylated proteins from *M. smegmatis* MC2 155. **(A)** Interaction analysis of Sauton-specific acetylated proteins. **(B)** Interaction analysis of 7H9-specific acetylated proteins. **(C)** Interaction analysis of medium-differential acetylated proteins.

Protein–protein interactions (PPI) form the foundation of cellular structure and function, with most proteins exerting their functions through interactions with others. Exploring these interactions can elucidate the physiological roles of acetylated proteins and enable the discovery of unknown protein functions through functional clustering (Li et al., 2025). Protein interaction analysis of the identified acetylated proteins was conducted using the STRING software for Sauton-specific (Figure 5A), 7H9-specific (Figure 5B), and medium-differential (Figure 5C) acetylated proteins. Concurrently, the obtained protein interactions were clustered using the MCODE plugin in Cytoscape.

**Figure 6:**
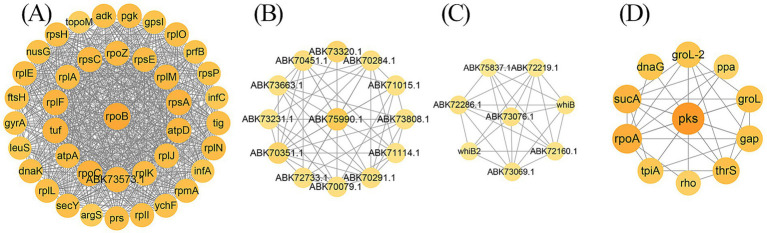
Clusters of Sauton-specific acetylated proteins. **(A)** Cluster 1: 40 nodes, 689 edges; MCODE score = 35.333. **(B)** Cluster 2: 13 nodes 51 edges; MCODE score = 8.500. **(C)** Cluster 3: 8 nodes 26 edges; MCODE score = 7.429. **(D)** Cluster 4: 11 nodes 34 edges; MCODE score = 6.800.

**Figure 7:**
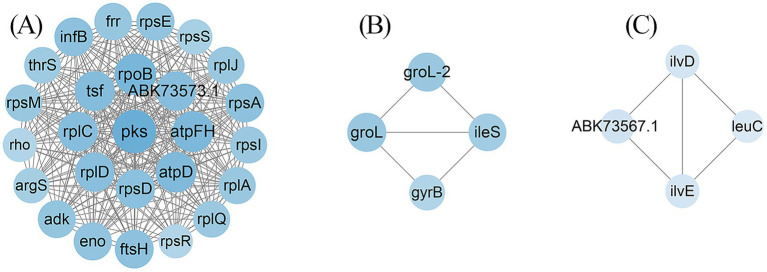
Clusters of 7H9-specific acetylated proteins. **(A)** Cluster 1: 26 nodes 296 edges; MCODE score = 23.680. **(B)** Cluster 2: 4 nodes 5 edges; MCODE score = 3.333. **(C)** Cluster 3: 4 nodes 5 edges; MCODE score = 3.333.

**Figure 8:**
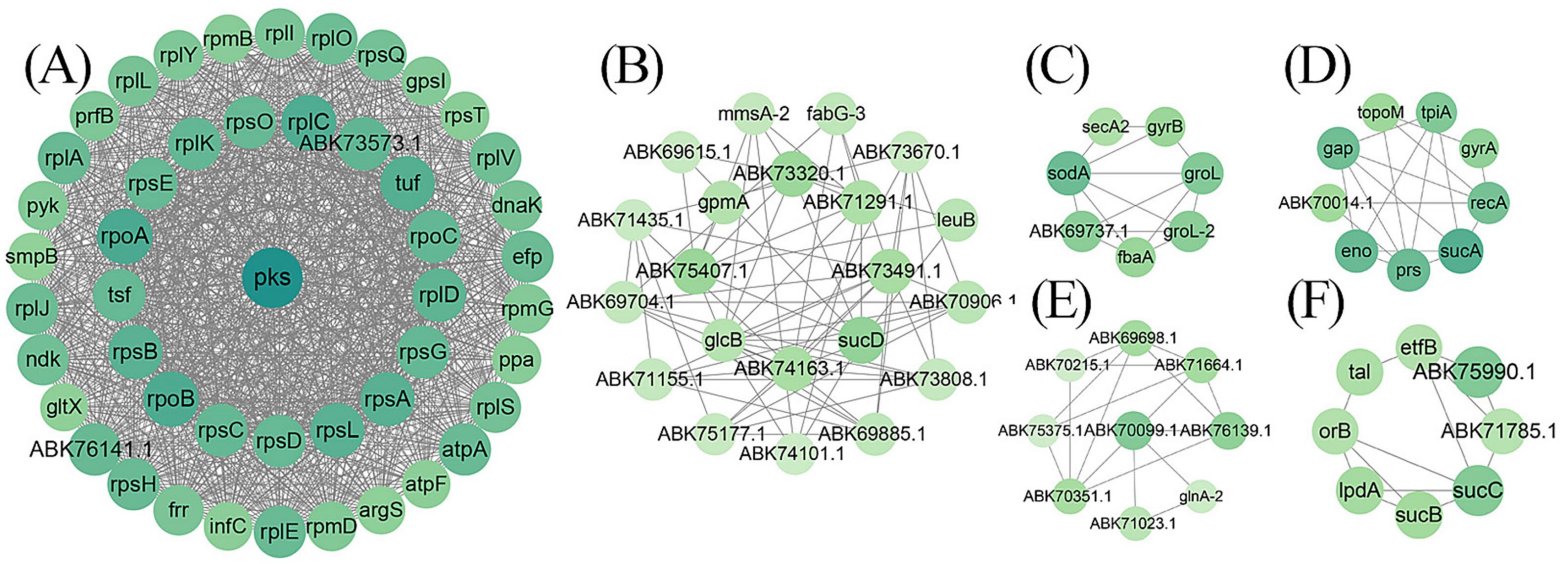
Clusters of medium-differential acetylated proteins. **(A)** Cluster 1: 48 nodes 1,041 edges; MCODE score = 44.298. **(B)** Cluster 2: 21 nodes 68 edges; MCODE score = 6.800. **(C)** Cluster 3: 7 nodes 14 edges; MCODE score = 4.667. **(D)** Cluster 4: 9 nodes 18 edges; MCODE score = 4.500. **(E)** Cluster 5: 9 nodes 16 edges; MCODE score = 4.000. **(F)** Cluster 6: 8 nodes 13 edges; MCODE score = 3.714.

Four clusters were identified among Sauton-specific acetylated proteins (Figure 6); the clusters primarily encompassed ribosomes, fatty acid metabolism, transcription, and RNA degradation. Three clusters emerged among 7H9-specific acetylated proteins (Figure 7), encompassing ribosomes, RAN degradation, and 2-Oxocarboxylic acid metabolism. Furthermore, six clusters were identified among medium-differential acetylated proteins (Figure 8), these involved ribosomes, biosynthesis of secondary metabolites, RNA degradation, amino acid biosynthesis, pyruvate metabolism, and carbon metabolism. Although proteins associated with RNA degradation were clustered in both Sauton-specific acetylated proteins, 7H9-specific acetylated proteins, and medium-differential acetylated proteins, the MCODE score was highest in Sauton-specific acetylated proteins, followed by medium-differential acetylated proteins.

### 3.5 Conservative analysis

**Figure 9:**
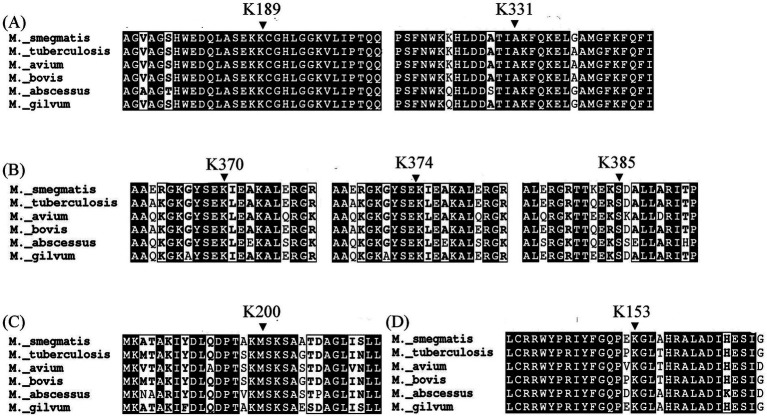
Conservative analysis. **(A)** Conservative analysis of ICL. **(B)** Conservative analysis of FabG. **(C)** Conservative analysis of TrpRS. **(D)** Conservative analysis of Orns.

As *M. smegmatis* was used in this experiment, we selected *M. smegmatis*, *M. tuberculosis*, *M. avium*, *M. bovis*, *M. abscessus* and *M. gilvum* to perform conservation analyses on proteins such as isocitrate lyase, 3-hydroxyacyl-CoA dehydrogenase, tryptophan-tRNA synthetase and oligonucleotidase, in order to extrapolate the results to *M. tuberculosis* (Figure 9). The conservation analysis indicates that lysine acetylation sites in *M. smegmatis* are highly conserved across the Mycobacterium genus, suggesting that acetylation at these sites is of significant importance to the genus.

## 4 Discussion

Mycobacteria can adapt to diverse nutritional environments within infected hosts, surviving under both nutrient-limited and nutrient-rich conditions. This remarkable adaptability is closely linked to their metabolic regulatory mechanisms. To investigate how lysine acetylation modifications in *Mycobacterium* proteins regulate cellular metabolism under differing nutritional conditions, we cultured *M. smegmatis* MC^2^ 155 in either Sauton or 7H9 media to simulate nutrient-limited and nutrient-rich environments, respectively (Zhang L. Y. et al., 2022). Bioinformatic analysis was then performed on the identified acetylated proteins.

The bioinformatics analysis revealed that lysine-acetylated proteins from bacteria cultured in Sauton medium exhibit a greater motif diversity than those from bacteria cultured in 7H9 medium. This effect may arise because, under nutrient-limited conditions, mycobacteria employ acetylation modifications to reduce the activity of relevant enzymes. This strategy ensures greater cellular participation in essential metabolic pathways while minimizing energy expenditure, thereby avoiding redundant pathways. Concurrently, the analysis of acetylated protein quantity and site prevalence indicated a significantly higher number of lysine-acetylated proteins and sites in bacteria cultured in Sauton medium compared with those cultured in 7H9 medium. This quantitative difference further supports the likelihood of the aforementioned hypothesis being valid.

KEGG analysis revealed that proteins involved in metabolic pathways, including the citrate cycle (TCA cycle), 2-oxocarboxylic acid metabolism, and carbon metabolism, were significantly enriched in bacteria cultured in 7H9 medium, with TCA cycle-associated proteins being the most prominent. Similar findings were reported by Liu et al. (2014) and Xie et al. (2015) in their study of *M. tuberculosis*. This finding indicates differences in carbon source metabolism between *M. smegmatis* MC^2^ 155 cultures grown in Sauton and 7H9 media. Based on current knowledge of mycobacterial metabolic characteristics under different nutritional environments, we hypothesize that *M. smegmatis* MC^2^ 155 cultured in 7H9 medium may preferentially utilize the TCA cycle for carbon metabolism, whereas culturing in Sauton medium may result in the activation of alternative pathways.

Regarding carbon utilization, mycobacteria differ from other bacteria in their capacity to simultaneously utilize multiple carbon sources to maximize growth (de Carvalho et al., 2010). 7H9 medium contains diverse carbon sources, facilitating the utilization of glycerol, glucose, oleic acid, and other substrates by mycobacteria. However, in Sauton medium, glycerol serves as the primary carbon source, thereby subjecting mycobacterial growth—particularly carbon metabolism—to stringent regulation. Consequently, in this experiment, the degree of acetylation of enzymes involved in carbon metabolism showed marked differences between the two media.

Isocitrate represents a pivotal node in the carbon metabolism processes of *M. tuberculosis*. Isocitrate can enter the glyoxylate pathway via isocitrate lyase (ICL), enabling the bacterium to adapt to nutrient-limited environments, while under the influence of isocitrate dehydrogenase (ICDH), it can enter the TCA cycle (Farnia et al., 2026). Research indicates that acetylation modification can reduce ICDH protein activity (Lee et al., 2017). This study identified five ICDH sites with higher acetylation signal intensity in bacteria grown in Sauton medium, with three additional sites showing acetylation exclusively in these bacterial strains. Conversely, ICL’s K189 exhibited higher acetylation signals in bacteria cultured in 7H9 medium (*p* < 0.05), and K331 was acetylated exclusively in these bacteria. The distinct acetylation patterns of ICDH and ICL are highly consistent with the known mechanisms by which *Mycobacteria* regulate carbon flux under different nutritional conditions. Therefore, we speculate that acetylation at the ICDH site may reduce ICDH activity, thereby shifting carbon metabolism from the key isocitrate node to the glyoxylate pathway to adapt to the nutrient-limited environment of Sauton medium. In contrast, acetylation at the K189 and K331 sites of ICL may reduce ICL enzyme activity, causing bacteria cultured in 7H9 medium to rely more heavily on the tricarboxylic acid cycle for carbon metabolism. Meanwhile, since both ICDH and ICL are regulated by negative regulators—in *M. tuberculosis* growing on glucose, ICL is negatively regulated by the RamB protein (Micklinghoff et al., 2009)—we hypothesize that, in addition to acetylation potentially reducing enzyme activity through direct protein inhibition, it may also decrease enzyme activity by affecting the binding of negative regulators.

Regarding energy expenditure, we found that under nutrient-limited conditions, *M. smegmatis* may reduce energy-intensive/non-essential metabolic processes, thereby channeling limited energy resources towards essential metabolic pathways for bacterial survival. 3-hydroxyacyl-CoA dehydrogenase (FabG) is a key enzyme in bacterial type II fatty acid synthesis (FASII) (Cross et al., 2021). This enzyme plays a crucial role in fatty acid metabolism and the synthesis of branched-chain fatty acid precursors. It belongs to the short-chain dehydrogenase/reductase (SDR) superfamily, which is involved in the NADPH-dependent addition or removal of hydrogen from specific substrates (Kim et al., 2025). Our findings demonstrate that the K370 site of FabG undergoes acetylation exclusively in bacteria cultured in Sauton medium. According to UniProt, this site is located within the NAD-binding domain, suggesting that it may potentially regulate enzymatic activity. Concurrently, K385 and K374 remain within this domain, though no significant differences were observed between bacteria cultured in either medium. Thus, we hypothesize that K370 acetylation may impair enzyme-NAD binding, leading to reduced FabG activity and, consequently, diminishing fatty acid synthesis—a process that demands substantial energy expenditure. The clustering of proteins involved in fatty acid metabolism within the protein interaction network among Sauton-specific acetylated proteins support this hypothesis. Whether this energy regulation occurs through FabG inactivation warrants further experimental verification. Protein synthesis, an energy-intensive metabolic process, may also be suppressed (Pereira et al., 2015). We observed acetylation at K200 (ATP-binding site) in tryptophan-tRNA synthetase (TrpRS), with increased acetylation signals in bacteria cultured in Sauton medium (*p* < 0.05). TrpRS catalyzes the synthesis of tryptophanyl-AMP from ATP and Trp, simultaneously producing Trp-tRNA^Trp^ by transferring Trp to tRNA^Trp^ (tRNA(Trp) + L-tryptophan + ATP = L-tryptophyl-tRNA(Trp) + AMP + diphosphate + H+) (Han et al., 2024). We found that K200 corresponds to the first Lys in the KMSKS sequence, and that the KMSKS sequence is highly conserved in both prokaryotic and eukaryotic TrpRS, particularly the first Lys (Chan and Koeppe, 1995). Current research has shown that alaninization of the first Lys in the KMSKS sequence of TrpRS in *E. coli* results in a 1,000-fold decrease in the enzyme’s catalytic rate (Chan and Koeppe, 1995); simultaneously, the KMSKS sequence in *Neisseria gonorrhoeae* contributes to the stability of the transition state in amino acid activation reactions, and mutations in this sequence may lead to antibiotic resistance and disruption of enzyme conformational dynamics (Baral et al., 2025). Thus, we propose a testable hypothesis that Acetylation of the ATP-binding site may exert a negative regulatory effect on TrpRS, thereby affecting the reaction tRNA(Trp) + L-tryptophan + ATP = L-tryptophyl-tRNA(Trp) + AMP + diphosphate + H+. This would result in reduced efficiency of ATP and Trp incorporation, thereby redirecting ATP and Trp to key reactions of greater importance to *Mycobacterium* under nutrient-limiting conditions.

We discovered that among the Sauton-specific and medium-differential acetylated proteins (higher signal intensity in Sauton medium), multifunctional oxoglutarate decarboxylase (SucA) exhibits acetylation at eight sites; sulphurtransferase (TBH48777.1) and inositol-3-phosphate synthase at seven sites; type I glyceraldehyde-3-phosphate dehydrogenase (GapA) at six sites; glucose-6-phosphate dehydrogenase (coenzyme-F420) (G6PD), phosphoglyceromutase (GpmI), and dihydrolipoyl dehydrogenase (Lpd) at five sites. All these proteins participate in carbon source metabolism. In this context, SucA is a component of the ketoglutarate dehydrogenase complex, a pivotal enzyme in the tricarboxylic acid cycle that catalyzes the conversion of *α*-ketoglutarate into succinyl-CoA. Acetylation may inhibit this enzyme’s activity, thereby redirecting greater quantities of α-ketoglutarate towards glutamate synthesis. This effect ensures an adequate nitrogen supply and balanced amino acid synthesis within Sauton medium, where asparagine serves as the nitrogen source. GapA and GpmI are core glycolytic enzymes responsible for converting three-carbon compounds produced during glycerol metabolism. G6PD and coenzyme F420, a mycobacterial-specific cofactor, participate in multiple redox reactions and confer resistance to oxidative stress. None of the 7H9-specific acetylated proteins exhibited five or more acetylation sites.

Moreover, unlike the 7H9-specific acetylated proteins identified in the KEGG analysis, which were found to participate in carbon metabolism, proteins associated with RNA degradation were significantly enriched among the medium-differential acetylated proteins. Concurrently, proteins involved in RNA degradation were clustered from both Sauton-specific acetylated proteins and medium-differentiated acetylated proteins in protein interactions, with numbers exceeding those in the 7H9 medium. Based on the aforementioned observations, we hypothesize that under nutrient-restricted conditions, *M. smegmatis* MC^2^ 155 may enhance its environmental adaptability by regulating RNA degradation. Diego et al. reported similar findings: under conditions of nutrient deprivation and hypoxia, *M. smegmatis* downregulates mRNA degradation mechanisms, thereby increasing mRNA stability (Vargas-Blanco et al., 2019). Oligoribonuclease (Orns), an RNA exonuclease involved in c-di-GMP degradation (Badhwar et al., 2022a), was found to undergo lysyl acetylation modification at position K153 exclusively in *M. smegmatis* MC^2^155 cultured in Sauton medium (according to UNIPROT, the acetylated K153 residue is located within the exonuclease domain). Mycobacteria have previously been demonstrated to exhibit elevated levels of the bacterial second messenger cyclic di-guanylate (c-di-GMP) under nutrient-limited conditions to ensure survival and adapt to restricted carbon (Badhwar et al., 2022b; Zhang J. et al., 2022). This elevated c-di-GMP promotes biofilm formation, conferring resistance to immune responses and antimicrobial treatments, leading to chronic and recurrent infections while enhancing bacterial virulence (Liu X. et al., 2024). Therefore, we hypothesize that acetylation at this site may reduce enzyme activity, thereby increasing c-di-GMP accumulation to adapt to nutrient-limited environments and promote bacterial resistance and virulence.

In summary, we analyzed proteome-wide lysine acetylation in *M. smegmatis* and identified metabolic pathways that undergo significant changes when the bacterium responds to environmental fluctuations. We also identified acetylation of several highly conserved proteins that are crucial for the bacterium’s adaptation to nutrient-limited conditions. We found that the bacterium regulates its metabolic pathways through acetylation to adapt to nutrient-limited conditions. With regard to carbon source metabolism, we found that enzymes such as ICDH and ICL in *M. smegmatis* exhibited differential acetylated expression under different nutritional conditions. In terms of energy metabolism, FabG, an enzyme associated with fatty acid synthesis, was expressed in an acetylated form only in Sauton’s medium; TrpRS, an enzyme associated with protein synthesis, showed enhanced acetylated expression in Sauton’s medium. With regard to RNA metabolism, the Orns was acetylated in *M. smegmatis* cultured in Sauton medium.

It should be acknowledged that this study has the following limitations. With regard to the differences in protein acetylation across different culture media, this paper merely speculates on the potential influences of a protein’s function, previous research, and bioinformatics results, without conducting experimental validation; this warrants further investigation in future studies.

## Data Availability

The original contributions presented in the study are publicly available. This data can be found here: https://www.iprox.org/, accession IPX0016996000 and PXD078081.

## Footnotes

1
http://www.genome.jp/kegg

2
https://psort.org/psortb/
